# What can we learn from a failed trial: insight into non-participation in a chat-based intervention trial for adolescents with psychosocial problems

**DOI:** 10.1186/1756-0500-7-824

**Published:** 2014-11-20

**Authors:** Rik Crutzen, Hans Bosma, Jano Havas, Frans Feron

**Affiliations:** Department of Health Promotion, CAPHRI School for Public Health and Primary Care, Maastricht University, P.O. Box 616, 6200 MD Maastricht, The Netherlands; Department of Social Medicine, CAPHRI School for Public Health and Primary Care, Maastricht University, Maastricht, The Netherlands; Youth Health Care Division, Regional Public Health Service GGD Zuid Limburg, Geleen, The Netherlands

**Keywords:** Non-participation, Chat-based intervention, Ehealth, Adolescents, Psychosocial problems, Youth health care

## Abstract

**Background:**

Psychosocial problems are highly prevalent among Dutch adolescents. We have conducted a trial to test whether offering chat-based consultations could be of added value within the context of Dutch Youth Health Care. This trial was ended prematurely because of recruitment issues. The aim of this study is to learn from this failed trial and to provide more insight into non-participation. Sources of data were non-participation forms, oral clarification, patient records, telephone interviews with adolescents that declined to participate, and a questionnaire-based process evaluation among nurses.

**Results:**

Non-participation appears to be a multi-factorial problem. Of those 290 adolescents eligible to participate, the majority (n = 165, 57%) declined to do so. Two-third of those (n = 109) also refused usual care, which might be indicative of not wanting help and/or experiencing problems and the validity of the assessment instrument. The other one-third (n = 56) did enrol in usual care and indicated other reasons for non-participation, such as a preference for face-to-face consultations, the extensive information that was provided, and not liking the idea of being randomized.

**Conclusions:**

This study shows that even if a trial fails, we can learn about the challenges of recruiting adolescents in intervention trials.

**Trial registration:**

NL37668.068.11/METC11-3-077.

## Background

Psychosocial problems are highly prevalent among Dutch adolescents: prevalence rates range from 19 to 28%, depending on socio-demographic characteristics (e.g., more likely among those with a low educational level [1]) and definition of psychosocial problems (e.g., behavioural problems, emotional problems) [2, 3]. These problems do not only impair current daily functioning [4, 5] and result in school drop-out [6], but are also known to track into adulthood [7] and lead to increased future health care costs [8]. However, the majority of adolescents do not receive professional help for their problems [9, 10], because they are often reluctant to seek professional help (e.g., due to shame) [11–13]. Therefore, future intervention initiatives should focus on removing barriers to seek help for psychosocial problems.

The use of the Internet as the primary delivery mode for interventions has expanded substantially [14], mainly due to a growth in access to the Internet, especially in high-income areas, such as the United States (78.6%) and Europe (63.2%) [15]. Interventions targeting psychosocial problems are delivered online nowadays, because it is known that adolescents feel empowered to talk about sensitive topics in such an online setting [16, 17]. Moreover, such interventions fit in with adolescents’ needs and use of the Internet [18, 19]. A qualitative study among Dutch adolescents confirmed the idea that they do feel the need to get information and help regarding their psychosocial problems via the Internet [20].

Intervening via chat could be a valuable opportunity, because approximately 90% of adolescents use the Internet to chat [21]. Chat refers to individual synchronous online chat counselling and therapy [22]. The use of chat in the context of an intervention can have a positive impact on outcome measures. Chat sessions led by mental health professionals, for example, resulted in a reduction of adolescents’ depressive complaints [23]. Another example, focusing on eating disorder prevention, showed that even a 1-hour moderated chat session about body image and eating significantly reduced eating pathology and improved self-esteem [24]. Moreover, shyness or anxiety does not pose an obstacle to use chat-based interventions and is even advantageous [25]. As concluded in a narrative review, chat-based interventions aimed at adolescents have a large potential public health impact [26].

Therefore, we have decided to investigate whether chat-based interventions could be of added value in the context of Dutch Youth Health Care (YHC). According to the Dutch Public Health Act, YHC Centres of the Regional Public Health Services (RHPS) have a statutory task to prevent and identify psychosocial problems among adolescents. As part of the YHC system, all children living in the Netherlands receive free preventive health examinations (PHE) regularly from birth through adolescence. The first PHE in adolescence takes place in the second year of secondary school (at the age of 13–14). If adolescents have psychosocial problems, then they are offered three additional face-to-face consultations with a YHC worker (in a time span of 3 months). We have conducted a trial to test whether offering chat-based consultations instead could be of added value, because adolescents might feel less shy and anxious to talk about their psychosocial problems.

The main reason to use YCH as a setting to conduct this trial was that this setting ensures access to adolescents, because assessing psychosocial problems and offering additional consultations are part of the regular procedure in PHE. In other words, the intervention is embedded in an existing setting, which is deemed beneficial [27]. Unfortunately, after almost 8 months of recruitment, only 12 adolescents participated (5 in the chat condition, 7 in the face-to-face condition) and the trial was terminated. This was not the first trial among youngsters that was ended prematurely because of recruitment issues [28]. Therefore, the aim of this article is to be able to learn from this failed trial, for future intervention initiatives, and to provide more insight into non-participation.

## Methods

To be able to learn from this failed trial, we (1) describe the trial procedure and (2) explain how we gained insight into non-participation from a point of view of both adolescents and YHC workers. The trial was a single-centre trial conducted at the RHPS Zuid Limburg (covering 18 municipalities). Adolescents were randomized to either the chat condition or the face-to-face condition. The Strengths and Difficulties Questionnaire (SDQ; as described later on) was used as primary outcome measure. Sample size calculations, assuming an alpha of .05 and a power of .90, resulted in 360 adolescents that had to be included at baseline.

### Ethics statement

The medical ethics committee of the University Hospital Maastricht and Maastricht University (METC azM/UM) granted ethical approval (Protocol ID: NL37668.068.11/METC 11-3-077).

### Trial procedure

First, the chat application was developed. The chat application was added to a separate website (i.e., not the website of the RHPS). Adolescents had to log on to this website, to make sure that it was only accessible to those who had agreed on a consultation with a YHC worker. Such agreements could be made directly, per phone, and per e-mail. The chat application was previously used by other organisations offering online support, which made sure that it was over its teething troubles. Subsequently, the YHC workers (i.e., nurses) were trained. A total of 19 nurses (i.e., all nurses at the RHPS Zuid Limburg) participated in this training. The training consisted of two days; the first day concerning online support in general, the second day focusing on online support by means of a chat-based intervention. It was an in-company training provided by an external partner with previous experience in offering online support. (Note: Additional meetings for nurses were planned to exchange their experiences regarding use of the chat application, but these were cancelled because the trial ended prematurely).

After these preparations, the actual recruitment started. This was kicked-off by a meeting for all nurses to explain the recruitment procedure and to provide them with all the materials needed (e.g., information letters, informed consent forms). Additional meetings were scheduled to discuss the disappointing number of recruited adolescents and the reasons for non-participation, and possible solutions. Based on these meetings, an incentive was raffled among participants (i.e., an iPad), the information letter for adolescents was shortened (from 3 pages to 1 page, with a link to a website containing the other information), and a YHC physician visited class rooms to explain the trial procedure before the PHE.

To assess psychosocial problems, adolescents had to fill in the self-administered Dutch version of the SDQ before the PHE, which is the common procedure. Furthermore, adolescents’ gender and educational level was registered (categorized as low – intermediate secondary education – and high – higher secondary education/preparatory university education). The SDQ has been validated among Dutch adolescents [29, 30] and its use is in line with national YHC guidelines [31]. The SDQ includes 25 symptom items and measures both negative (difficulties) and positive (strengths) behavioural and emotional attributes of the adolescent. A total score of 12 or higher on the SDQ is a proxy for an adolescent having psychosocial problems [32] and this cut-off is used as the primary inclusion criterion for the trial. Secondary exclusion criteria, which were only assessed if an adolescent met the first inclusion criterion, were adolescents already receiving treatment for their psychosocial problems (e.g., by a psychologist) and adolescents attending special education.

If adolescents were eligible to participate, they were invited to participate in the trial during the PHE. In line with ethical guidelines, they were offered one week time for reflection regarding this decision. Moreover, they were given an information letter and informed consent form, as well as a non-participation form if they decided not to participate. Adolescents were also given an information letter and informed consent form for their parents. Ethical guidelines require that both the adolescents as well as their parents sign the informed consent form. After one week, adolescents were contacted again to confirm whether they agreed to participate and to remind them about the non-participation form. Subsequently, adolescents were randomized and a consultation was scheduled based on the condition they were assigned to (i.e., face-to-face or via chat).

### Assessing non-participation

We had initially planned to conduct a questionnaire-based process evaluation among those adolescents using the chat-based intervention. We cannot draw strong conclusions based on these data due to the limited number of participants in the chat condition (n = 5). Therefore, we have decided instead to focus on the non-participation among adolescents. Four sources of data were used to gain more insight into possible reasons behind this non-participation from the point of view of adolescents.

#### Non-participation form

Both adolescents and their parents could fill out a non-participation form with predefined categories. Categories were, for example, the trial being too burdensome, not seeing the added value of the trial, and no preference for online support.

#### Oral clarification

Adolescents’ remarks about the trial that were made to the nurse during the PHE and phone calls from parents afterwards were collected as field notes. This was non-standardized information.

#### Patient records

Adolescents’ patient records were examined to ascertain whether they enrolled in usual care (i.e., whether appointments were made for face-to-face consultations) despite declining to participate in the trial.

#### Telephone interviews

A brief telephone interview was conducted with a random sample of adolescents that declined to participate, but did enrol in usual care. The main open-ended question asked to those adolescents was what their reasons were to decline participation in the trial.

The first source of data (i.e., the non-participation form) was planned *a priori*, all the other sources of data were used after the trial was terminated. Besides the point of view of adolescents, we have also decided *a posteriori* to pay extra attention to non-participation in the planned questionnaire-based process evaluation among nurses (n = 19). We have added open-ended questions regarding how to improve information regarding the trial (e.g., to nurses, to parents, to adolescents) and how to improve the recruitment procedure.

## Results

### Recruitment in the trial

**Figure 1:**
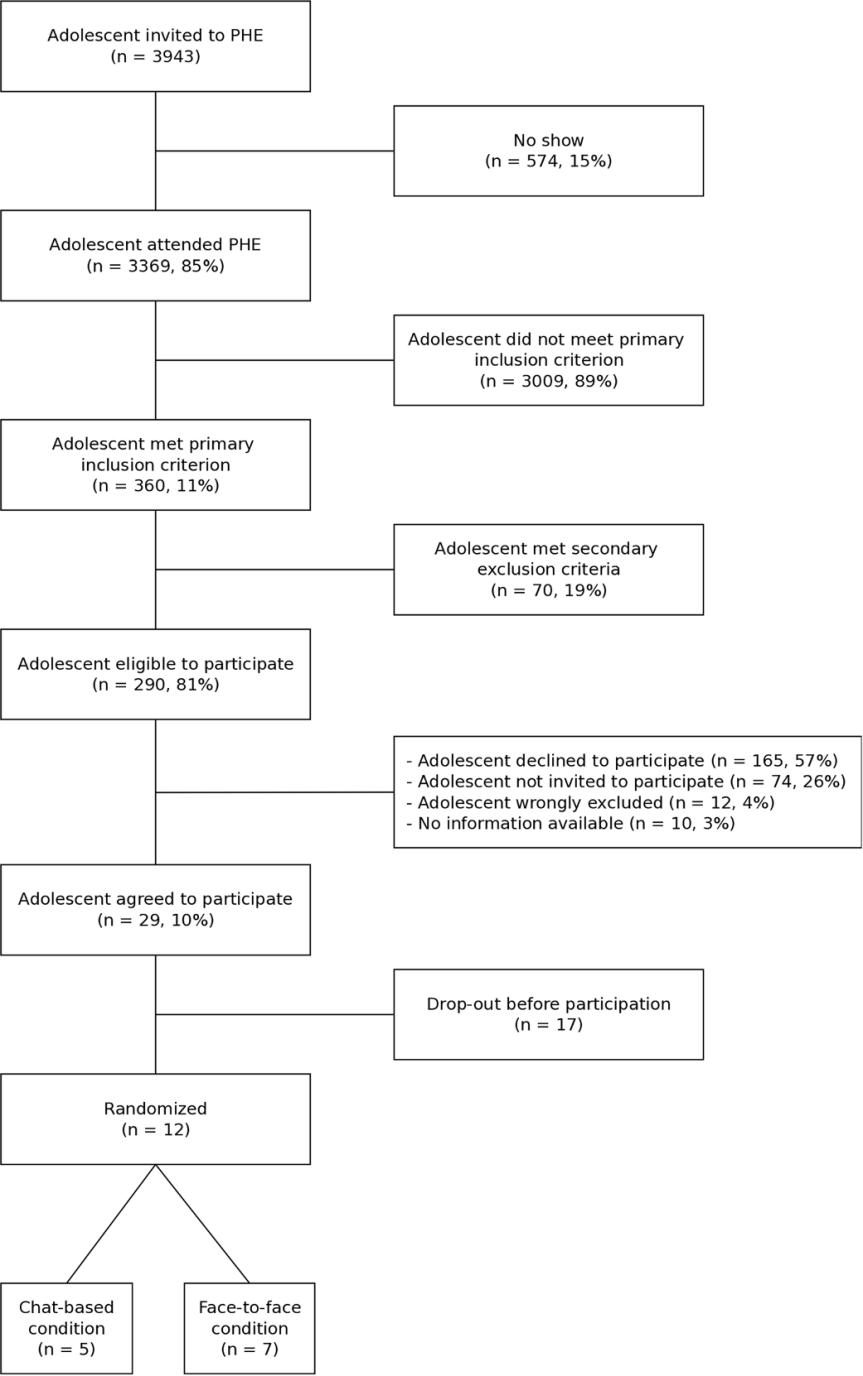
**Flowchart of recruitment in trial.**

Recruitment lasted from April 1^st^, 2012 until November 24^th^, 2012. Figure 1 shows the flowchart of recruitment in the trial. A total of 3,943 adolescents were invited to the PHE, of whom 574 did not show up (15%). Of the remaining 3,369 adolescents that attend the PHE, 360 adolescents scored 12 or higher on the SDQ (11%) and 290 of them were eligible to participate. Of those eligible to participate, 74 adolescents were not invited to participate (26%) and 12 adolescents were wrongly excluded (4%). There was no information available regarding 10 adolescents (3%). A majority of 165 adolescents (57%), however, declined participation. Adolescents that declined to participate did not differ in terms of gender (χ^2^ = 0.10, *p* = .75) or total score on the SDQ (t(288) = 0.89, *p* = .38), but declining to participate was more likely among those with a high educational level (χ^2^ = 5.96, *p* = .02). The reasons behind this non-participation were identified by means of data triangulation from several sources (as described in the Methods section).

### Non-participation

Of those 165 adolescents declining to participate, only 15 completed a *non-participation form* as well as 8 of their parents. Although these results cannot be generalized, a few of them (i.e., 4 out of 15) indicated that they did not prefer online support; 2 out of 8 parents mentioned the same reason. The *patient records* revealed that the decision not to participate was made mainly by adolescents themselves (n = 152; 92%) and only to a limited extent by their parents (n =13, 8%). Fifty-six adolescents (34%) that declined participation in the trial, enrolled in usual care. Oral clarifications revealed limited additional information regarding this group. Nevertheless, 109 adolescents (66%) did not only decline to participate in the trial, but did also refuse the offer for additional consultations (i.e., usual care). *Oral clarifications* indicated reasons such as “*does not experience problems*”; “*will call if help is needed*”, and “*does not see added value of further intervention by YHC*”. A random sample of 17 adolescents that declined participation in the trial, but did enrol in usual care, were invited for a *telephone interview*. A total of 13 adolescents agreed to take part in this interview (response rate: 76%). Some (n = 3) mentioned being randomised as a reason for non-participation, while others (n = 2) had no objection to this. Some (n = 5) preferred face-to-face consultations, while others (n = 3) preferred chat consultations.

All 19 nurses indicated in the *questionnaire* that it was clear to them that the trial concerned a chat-based intervention for adolescents that scored 12 or higher on the SDQ. Thirteen nurses indicated that information given to the parents could be improved; e.g., “*the letter to the parents was quite scary, as if we assumed that there were psychological problems*”. A suggestion, for example, was to provide “*a clear and compact flyer describing the inclusion criteria and steps within the project*”. With regard to information given to the adolescents, it was suggested to shorten the information letter and to provide more information in the class room (both suggestions were implemented during the trial). Some remarks were made about using the SDQ-score as an inclusion criterion. Regarding high SDQ-scores, nurses reported e.g., “*this can usually be explained by characteristics of reaching puberty*”; “*this does not always mean that there are problems*”. Nurses mentioned the extensive information and signing of informed consent forms as the main reason for non-participation. Another reason, according to the nurses, was a preference for face-to-face consultations or “*online support, but preferably really anonymous*”.

## Discussion

Non-participation in a chat-based intervention trial for adolescents with psychosocial problems appears to be a multi-factorial problem, which does not only depend on trial procedures (e.g., information letters, randomization). On the one hand, staff adherence might have been a problem. Despite a kick-off meeting to explain the recruitment procedure and to provide all the materials needed to nurses, adolescents were not invited to participate, were wrongly excluded, or there was no information available about their status in the trial. It is important, therefore, to constantly monitor staff adherence to the trial procedure. Moreover, recruitment via research staff might come with an additional cost when compared with practitioners (e.g., nurses), but this might improve recruitment [33]. On the other hand, nurses and adolescents reported several other issues. The focus here is to explore non-participation among the majority of adolescents that was eligible to participate, but declined to do so.

Two-third of those that declined to participate in the trial also refused usual care. This might be because they do not *want* help for their psychosocial problems by YHC or other mental health professionals or they do not *experience* problems [34]. A systematic review revealed barriers that adolescents perceive to seek help. The main barriers are stigma and embarrassment, problems recognising symptoms (i.e., poor mental health literacy), and a preference for self-reliance [35]. Therefore, future intervention strategies should address adolescents’ desire for self-reliance (e.g., by providing evidence-based self-help material) and should aim to reduce stigma associated with ‘mental illness and mental health help-seeking’. Incentives do not always encourage participation, as this also depends on the information provided about the intervention itself [36], especially for those who experience problems. With regard to recognising symptoms, it is important to increase adolescents’ mental health literacy. These suggestions by Gulliver and colleagues [35] are all preventive in nature and are relevant for all adolescents.

The high percentage refusing usual care as well might also raise questions about the validity of the self-administered version of the SDQ for adolescents, as they might score 12 or higher but still not experience problems. Although the SDQ was not initially designed for this purpose, it has become an integral component of assessment packages for service and research purposes (e.g., as an outcome measure). Nevertheless, the use of the SDQ alongside a number of other tools is the preferred method of its use [37]. It is recommended, for example, to complete such assessments with evaluations by parents and teachers [38]. The high specificity of the SDQ and its strong associations with psychiatric disorders make it a good indicator of whether additional clinical assessment and intervention may be necessary. A previous study concluded that “the SDQ may have maximal value as part of a routine assessment in high-risk paediatric clinics based on perceived difficulties by parents, teachers, or children. While improving the efficiency of case-finding, the burden of medical cost and potential distress caused by false positives can also be significantly reduced” [39]. It is worth noting that declining to participate was more likely among those with a high education level. It is unclear why this is so, as non-participation is generally higher in the lower educated groups [40], where problems are generally more severe and needs for intervention accordingly higher [1]. It might be that those with a high educational level are more inclined to use private services and by-pass RPHS. Focusing on differences between groups in terms of non-participation is an interesting avenue for further research.

The other one-third of those that declined participation in the trial did enrol in usual care. Some of those preferred face-to-face consultations, which might be because counselling is still viewed as an interpersonal experience [41]. This was not expected, however, based on previous findings among this target group indicating that they do feel the need to get information and help regarding their psychosocial problems via the Internet [20]. Being anonymous is one of the reasons why people prefer online support [14], but as adolescents were already in a face-to-face setting with a nurse, this feeling of anonymity might have been less in the study at hand. We think it is safe to conclude, however, that chat-based consultations should merely be an addition that is appropriate for some adolescents and not a replacement of usual care (i.e., face-to-face consultations) for all adolescents.

Other reasons for non-participation in the trial were the information letter that was provided and adolescents that did not like being randomized. We tried to improve the information letter, but were limited in the sense that the medical ethics committee requires certain information to be given to adolescents and their parents. A previous study testing strategies such as providing additional information and direct mailing to the parents did not find any differences in terms of improved consent rates [42]. It might be worthwhile to explore a two-stage recruitment process with a youth-friendly invitation before the official information letter. Another study indicated that adolescents became confused by being presented with options (e.g., chat-based consultations) that were then removed by randomisation [43]. A Zelen randomised consent design where eligible participants are randomly allocated prior to being approached about the trial could be considered in future research [44]. So, consent can be sought conditionally (i.e., for the condition an eligible participant is assigned to), without the uncertainty of randomization.

A limitation of this study is that the decision to use certain sources of data was often made *a posteriori*. This is in the nature of this study, as its aim was determined after the trial was terminated. Moreover, non-participation in the trial is also likely to result in less willingness to complete non-participation forms and reluctance to participate in telephone interviews concerning non-participation. This resulted, in the current study, in relatively small samples for these sources of data. Although additional efforts (e.g., extra phone calls) might result in larger samples, one may also wonder whether this results in ethical conflicts if adolescents already reported that they did not want to participate in the trial. Nevertheless, it might be wise for future trials to use such additional sources of data, even if the trial is successful in terms of recruitment. Moreover, non-participation forms (completed by those refusing to participate) and patient records (completed by staff) can also be routinely checked during the recruitment phase to detect problems at an early stage. Future research might also include adolescents that do participate to assess ‘reasons for participation’ besides ‘reasons for non-participation’. This requires a “comprehensive data monitoring system with strong administrative support at both central and local levels” [45]. The issue of non-participation is also important outside the context of a trial. Many interventions that prove efficacious in trials are much less effective when disseminated outside the trial context [46]. It has been argued that much efficacy research is conducted in such a way that it “decontextualizes” an intervention effect by, for example, studying narrowly selected participants or using intervention strategies that are hard to implement by typical intervention agents [47]. This has been acknowledged by the field, as indicated by an increase in adoption and implementation research [48].

## Conclusion

This study shows that even if a trial fails, we can learn about the challenges of recruiting adolescents in intervention trials. Non-participation is a multi-factorial problem and is amongst others related to a preference for face-to-face consultations, the extensive information that was provided, and not liking the idea of being randomized. Moreover, adolescents do not necessarily *want* help for their psychosocial problems or they might not *experience* problems.

## Abbreviations

YHC: Youth health care
RHPS: Regional public health service
PHE: Preventive health examinations
SDQ: Strengths and difficulties questionnaire.
